# Climate Change and Children’s Mental Health: A Developmental Perspective

**DOI:** 10.1177/21677026211040787

**Published:** 2021-09-14

**Authors:** Francis Vergunst, Helen L. Berry

**Affiliations:** 1 Department of Social and Preventive Medicine, University of Montreal; 2 Ste-Justine University Hospital Research Center, Montreal, Québec, Canada; 3 Faculty of Medicine and Health, University of Sydney; 4 Australian Institute of Health Innovation, Macquarie University

**Keywords:** developmental psychopathology, psychiatry, climate change, global warming, disasters, inequality, birth cohort, administrative data, long term

## Abstract

Climate change is a major global public-health challenge that will have wide-ranging impacts on human psychological health and well-being. Children and adolescents are at particular risk because of their rapidly developing brain, vulnerability to disease, and limited capacity to avoid or adapt to threats and impacts. They are also more likely to worry about climate change than any other age group. Drawing on a developmental life-course perspective, we show that climate-change-related threats can additively, interactively, and cumulatively increase psychopathology risk from conception onward; that these effects are already occurring; and that they constitute an important threat to healthy human development worldwide. We then argue that monitoring, measuring, and mitigating these risks is a matter of social justice and a crucial long-term investment in developmental and mental health sciences. We conclude with a discussion of conceptual and measurement challenges and outline research priorities going forward.

Climate change is a global public-health emergency. The World Health Organization (WHO) described it as “the greatest challenge of the 21st century, threatening all aspects of the society in which we live” (Campbell-Lendrum et al., 2018, p. 8), and the *Lancet* countdown on climate change noted that unless urgent and vigorous action is taken, “trends in climate change impacts, exposures, and vulnerabilities demonstrate an unacceptably high level of risk for the current and future health of populations across the world” (Watts et al., 2018, p. 1861). The worst effects can still be avoided by limiting the average global temperature rise to 1.5 °C through rapid greenhouse-gas reductions that include reaching net zero by 2050. A partial response, characterized by moderate emissions reductions, will produce temperature rises between 2.1 °C and 3.5 °C by 2100, leading to profound disruptions to earth systems and human societies (Intergovernmental Panel on Climate Change, 2021; Steffen et al., 2018).

The impacts of climate change on physical heath are now well documented (Berrang-Ford et al., 2021; Rocque et al., 2021), but mental health is also being affected (Charlson et al., 2021; Lawrence et al., 2021). Hotter average temperatures and more frequent and severe heatwaves are linked with increased population-level psychological distress (Ding et al., 2015; Thompson et al., 2018), self-harm (M. N. Williams et al., 2016), hospital psychiatric admissions (Basu et al., 2018; Wang et al., 2014), and suicide (Burke et al., 2018; Obradovich et al., 2018). Heat waves aggravate existing mental disorders (Noelke et al., 2016), especially in conjunction with high humidity (Mora et al., 2017), and reduce the effectiveness of certain psychotropic medications (Cusack et al., 2011). The statistical effect size for the impact of hot days on population mental health has been observed to be equivalent to that of unemployment (Ding et al., 2015). Extreme water stress already affects a quarter of the world’s population (Hofste et al., 2019) and is expected to increase with global heating, which would aggravate famine, civil conflict, forced migration, and war (Hoffmann et al., 2020). An estimated 22.5 million people have been displaced by climate- or weather-related disasters each year for the past 7 years (Internal Displacement Monitoring Centre, 2021), increasing mental health vulnerability (Siriwardhana & Stewart, 2013).

More frequent, unpredictable, and intense extreme weather events—such as storms, floods, and wildfires—are a well-documented consequence of global climate change (Watts et al., 2020; World Meteorological Organization, 2020). They destroy homes, livelihoods, and vital infrastructure and can lead to psychological distress, anxiety, depression, and posttraumatic stress disorder (PTSD) in adults and children (Matthews et al., 2019; McDermott et al., 2014; Norris et al., 2002; O’Brien et al., 2014). Disasters can exhaust inadequate and already overstretched mental health services and reduce access to services and medications, whereas incremental changes, such as rising sea levels, disrupted weather patterns, and loss of agricultural and ancestral lands, lead to homelessness, food shortages, and displacement (Black et al., 2011). In August 2019, the first ever national survey of the mental health impacts of climate change reported that residents of Greenland were experiencing unprecedented levels of stress and anxiety about their changing landscapes and climate (Minor et al., 2019). The term *solastalgia* has been increasingly employed in the climate-change and mental-health literature to describe “distress or desolation caused by the gradual removal of solace from the present state of one’s home environment.” (Albrecht, 2011, p. 50).

The effects of climate change on mental health will be unequally distributed both within and between nations. Certain geographical regions (e.g., equatorial, Arctic) and vulnerable populations, such as socially isolated groups (Berry et al., 2008), Aboriginal communities (Cunsolo & Ellis, 2018; Furgal & Seguin, 2006; Rigby et al., 2011), and women and children, will be most at risk (Carnie et al., 2011; Nelson et al., 2002). This is because climate change operates as a risk multiplier (Berry et al., 2018; McMichael, 2013) and compounds risks for already vulnerable populations, which have more limited capacity to adapt to or to avoid new threats and impacts (Norris et al., 2002). Climate change will be a key driver of existing social and health inequalities, and tracking and mitigating these effects is therefore a matter of social justice (Sanson & Burke, 2020).

## A Developmental Perspective

Although the body of literature linking climate-change effects to mental-health risk is growing, much less has been reported about risks to children. This is surprising because childhood is a period of extremely high developmental vulnerability when most psychiatric disorders are first established (Paus et al., 2008; Solmi et al., 2022). So far, there has been limited systematic theoretical or empirical work examining the pathways and processes linking climate-change-related stressors and mental-health vulnerability in children and adolescents, which is urgently needed so that effective adaptative and mitigative strategies can be implemented. These challenges, we argue, are ideally studied from a developmental life-course perspective.

Developmental approaches are not new to the mental-health sciences. They emerged under the banner of developmental psychopathology in the 1970s (Achenbach, 1974), were strongly advanced in the interdisciplinary psychiatric research of the 1980s and 1990s (Rutter, 1988), and continue to be called for by clinicians and researchers today (Jaffee, 2019; Thapar & Riglin, 2020). Developmental psychopathology takes an expansive view of the processes and pathways that lead to normal and abnormal development (Eme, 2017), integrating multiple areas of psychology and related disciplines relevant to child psychopathology and developmental sciences (Cummings & Valentino, 2015). The approach is predicated on the observation that most disorders begin early in life; that they are the consequence of physiological, neurobiological, and behavioral phenotypes that change across time and in response to environments; and that the severity and timing of exposure to risks have lifelong effects on outcomes (Baram et al., 2012; Lupien et al., 2009).

Developmental approaches maintain that normal and abnormal developmental processes are mutually informative and should therefore be considered together (Eme, 2017). No prior assumptions are made about the question of continuities and discontinuities between normality and abnormality, and mental-health problems are viewed as quantitative dimensions rather than as qualitative categories (Martin et al., 2018). In this article, we take a broad view of mental health that is not limited to psychopathology or circumscribed psychiatric diagnoses but includes healthy cognitive, behavioral, and emotional development, which are known to covary with mental-health risk, as well as states of mental health, resilience, and well-being (Hayes et al., 2018). Put another way, good mental health can be understood as a dynamic adaptive capacity that incorporates the ability to regulate emotional and mental states in the face of life’s normal ups and downs (Herrman, 2001).

Developmental approaches have been strongly shaped by epidemiological methods such as birth-cohort studies, which have been instrumental in elucidating the causal processes and mechanisms of many psychiatric disorders (Rutter, 1988). It is this developmental literature, with its focus on longitudinal and particularly birth-cohort designs, that we draw on in this article. A complete analysis of the key conceptual elements of developmental psychopathology, including emerging directions and topics of debate, can be found elsewhere (Beauchaine et al., 2018; Cummings & Valentino, 2015).

**Fig. 1.**
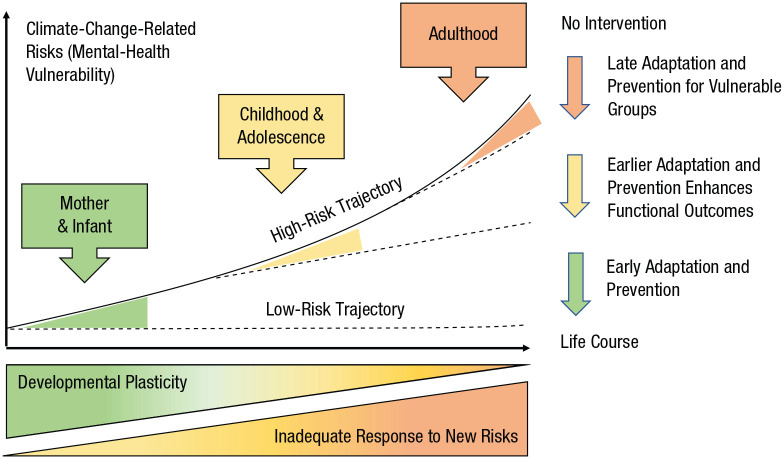
The effect of timing of adaptation and prevention on mental-health vulnerability in the context of climate change. Mental-health vulnerability increases in a nonlinear way through exposures that operate with additive, interactive, and cumulative effects (e.g., extreme weather events, heat exposure, worry about climate change). At the same time, plasticity decreases, and risk of exposures to new threats rises with time and because of unmitigated climate change (green-yellow triangle and yellow-orange triangle). Adaptation and prevention efforts (e.g., effective disaster-response planning, climate-change education) that begin early are more successful at reducing mental-health risk (low-risk trajectory) compared with efforts that begin in adolescence or adulthood (high-risk trajectory), especially for vulnerable populations. Adapted under Creative Commons license from Baird et al. (2017) and with permission from Hanson and Gluckman (2014).

A key rationale for applying a developmental framework to climate-change research concerns adaptation and prevention. Figure 1 shows that adaptation and prevention efforts in the early years (green arrow) will have much larger effects on the long-term psychological health and well-being of children than programs targeting them in adolescence or adulthood (yellow and orange arrows, respectively), when disorders are already established. One reason for this is that early life exposures to adversity operate with additive, interactive, and cumulative effects that place children on developmental trajectories leading to increasing mental-health vulnerability (Beauchaine et al., 2018). Developmental approaches provide a framework for conceptualizing multiple interacting risks across development and help identify specific populations and phenotypes that can be targeted with support, prevention, and intervention programs.

In this article, we have two objectives. First, we describe how a developmental perspective can inform and structure current thinking around the effects of climate change on psychological health and well-being from the perinatal period to adulthood. Second, we aim to advance thinking on how these threats can be conceptualized, measured, and prioritized. Specifically, we aim to provide an initial framework that can guide research, policy development, and intervention planning. In addition, we offer tentative recommendations indicating a way forward for new empirical research that can improve measurement of climate-change threats related to mental health. Note that studies reviewed in this article were based on samples that were more likely to come from high-income countries and to be majority White, although they were largely balanced by sex.

## A Note on Causal Pathways

It is generally agreed that climate change will not lead to the creation of new classifications of psychiatric disorders—although terms such as *eco-anxiety* have emerged as new variations on anxiety (Cunsolo & Ellis, 2018)—but instead will exacerbate established risk factors for already known disorders (McMichael, 2013). These effects are expected to occur through several pathways, including (a) acute events such as storms, floods, and wildfires; (b) subacute or long-term changes such as droughts and heat stress; and (c) long-lasting changes to landscapes and physical environments caused by rising sea levels and altered ecosystems and landscapes (Berry et al., 2010; Palinkas & Wong, 2020). These effects can increase mental-health vulnerability through direct pathways (e.g., severe weather event) and indirect pathways (e.g., increased physical health burden, social and economic disruptions, forced migration; Berry et al., 2018; Box 1).

**Box 1**

**Direct and Indirect Pathways Leading to Increased Mental-Health Risk**

**Direct (proximal) threats**

**Weather events (acute, subacute, and chronic)**

Exposure to the effects of weather and changing climate systems. These include hotter temperatures and extreme weather events—such as heatwaves, wildfires, droughts, storms, and floods—which can exacerbate existing mental problems, lower the threshold for relapse, or increase risk for the onset of new disorders.

**Existential threat**

Worry, anxiety, anger, and frustration about the effect of climate change, including the current and anticipated destruction and loss of landscapes, biodiversity, ecosystems, traditional lands, and special places. These threats can aggravate existing symptoms or lower the threshold for the onset of new mental-health disorders.

**Indirect (distal) threats**

**Physical health**

Injury, chronic diseases, the spread of vector-borne infectious diseases such as malaria, increased air pollution, and extended allergy seasons, which interact with mental-health vulnerability.

**Systemic instability and change**

Social and economic disruptions including the effects of water scarcity, reduced agricultural yields, famine, civil unrest, forced displacement, and war.

Establishing causality of climate-change-related exposures on mental health is challenging. In general, acute events such as hurricanes and wildfires lead to widespread damage and destruction and have intuitively self-evident and relatively immediate effects on mental health and well-being. The causal pathways are therefore more easily observed, understood, and measured. But the causal effects of subacute and long-lasting exposures on mental health are much harder to establish. This is because these exposures may be numerous, diffuse, variable in intensity and duration, and temporally disassociated, so that identifying and disentangling their unique contribution to mental-health risk becomes difficult. The direct and indirect pathways leading to increased mental-health risk therefore present an important theoretical and measurement challenge (see Box 2).

**Box 2**

**Attributing Mental-Health Problems to Climate Change**

*Climate change* describes a complex set of phenomena that include, among other effects, major changes in underlying global surface and, especially, sea temperatures, precipitation, and wind patterns that occur over decades or longer. Humans inhabit a relatively narrow climate niche with intense local adaptation; changes to weather and climate systems can upset these adaptations, causing widespread disruptions to established systems. Attributing mental-health problems to these disruptions is challenging. Mental-health problems are complex disorders that are shaped by the interplay of biological, psychological, social, and environmental factors that are the result of long and intricate causal chains that begin before birth and extend across development (Berry et al., 2018). Linking complex outcomes back to single events or even a series of events is therefore problematic because many questions about timing, severity, and chronicity of exposure remain. Nevertheless, growing empirical and theoretical work indicates that climate-change-related threats—including acute (e.g., floods, wildfires), subacute (e.g., protracted heat stress), and chronic (e.g., drought) events—are causally associated with increased mental-health risk (Lawrance et al., 2021; Watts et al., 2018, 2020; Charlson et al., 2021). Furthermore, the effects of these different kinds of exposures appear to be linked with increased risk for different kinds of mental-health outcomes; for example, slow-creeping weather-related events such as protracted droughts are more strongly associated with mood disorders, and acute events such as floods are associated with anxiety disorders.

Another question concerns whether fear, anxiety, worry, and despair about climate change are causing an increase in common disorders such as anxiety (Berry & Peel, 2015). Climate change poses real and significant threats to human health and well-being, so some worry about its effects is clearly justified (Cunsolo et al., 2020). But distinguishing “normal” worry from pathological anxiety can be difficult (Clayton, 2020). On the one hand, as Hayes and colleagues (2018) noted, there is a risk of pathologizing psychological responses to transient adverse life events and thus misattributing these to climate change, whereas on the other hand, there is a risk of failing to recognize how climate change may be affecting mental health. For instance, no studies have yet considered whether a hypothesized climate-change-related anxiety disorder would have presented anyway, triggered by another cause (e.g., worry about a lethal global pandemic or nuclear war), in the absence of climate change. Nor have studies examined whether background worry about climate change is increasing the likelihood that *any* psychiatric disorder may present. It may be that although climate change does not cause the widespread onset of mental-health problems, it exacerbates symptoms for those with preexisting morbidity (i.e., provides an additional substantive stressor), or adds new dimensions to their psychopathology (e.g., adds “eco-anxiety” to an existing anxiety disorder), or both (Berry & Peel, 2015).

## Climate Change and Children’s Mental Health

Young people today make up 41% of the global population; 25.4% are 0 to 14 years old, and 15.5% are 15 to 24 (United Nations, 2020). The United Nations Children’s Fund (2021) estimates that nearly half of the world’s 2.2 billion children are now at “extremely high risk” from the impacts of climate change. More than 88% of the current burden of disease attributable to climate change occurs in children younger than 5 (Sheffield & Landrigan, 2011). Climate change is predicted to worsen all top five causes of infant mortality, including malnutrition, neonatal deaths, acute respiratory illness, diarrhea, and malaria. Compared with adults, children have less effective heat-adaptation capacities, have higher exposure to toxins per unit of body weight (e.g., water-, air-, and food-borne), are more vulnerable to insect-borne vectors, and have more life years ahead in which to be exposed to worsening and new climate-change threats (Colón-González et al., 2021; Garcia & Sheehan, 2016; Sheffield & Landrigan, 2011). Moreover, 85% of the world’s children live in developing countries (United Nations Children’s Fund Office of Research, 2014), which are most vulnerable to climate-change threats because of geographical location and resource limitations that make it more difficult to adapt (Harville et al, 2010; Bathiany et al., 2018). Physical health problems, including noncommunicable disease risk (Olson & Metz, 2020), contribute to the global mental-health burden because they interact with psychopathology risk and increase the personal, social, and economic burden of mental illness (Firth et al., 2019).

Children and adolescents already bear a large mental-health burden. Epidemiological studies show that in the United States, nearly 50% of teens ages 13 to 18 years meet *Diagnostic and Statistical Manual of Mental Disorders* criteria for at least one disorder, and 27.6% meet criteria for a “severe disorder” (Merikangas et al., 2010); one half of affected children receive no treatment (Whitney & Peterson, 2019). A recent meta-analysis of 192 epidemiological studies worldwide showed that the proportion of individuals who experienced the onset of any mental disorder before the ages of 14, 18, and 25 years was 34.6%, 48.4%, and 62.5% respectively. The peak age of onset for any mental disorder was 14.5 years (Solmi et al., 2022). Note that because of the large burden of infectious disease, prevalence estimates for mental disorders appear marginal in many developing countries (Cortina et al., 2012) but are expected to rise as these populations follow the socioeconomic shifts already observed in high-income countries over the 20th century. The global mental-health burden for children is therefore likely to increase in the years ahead (Baranne & Falissard, 2018).

Mental-health problems disrupt young people’s educational and occupational opportunities; increase stigma, discrimination, and social marginalization; and are associated with increased health burden, higher lifetime suicide risk, and earlier mortality (Corrigan & Watson, 2002; Mojtabai et al., 2015; Liu et al., 2017). As climate change undermines children’s mental health, it increases this global burden (Burke Sanson & Van Hoorn, 2018; Cianconi et al., 2020; Clemens et al., 2022; Evans, 2019). In the following section, we apply a developmental lens to examine the pathways and processes through which these mental-health impacts occur.

## A Developmental Perspective on Climate-Change Risks

A core assumption of developmental psychopathology is that development is influenced by physiological, genetic, cognitive, emotional, social, and environmental factors and the dynamic interplay between them (Beauchaine et al., 2018). Ecological models provide a framework for conceptualizing this dynamic interplay. Risks are understood as an interaction between individual processes situated within the context of families, communities, and societies (Noffsinger et al., 2012). Thus, in the context of climate-change risk, healthy human development can be disrupted at multiple levels: biological (e.g., stress-induced changes in the stress response system, changes to DNA methylation), microsystems (e.g., increased family stress, reduced quality of parenting), mesosystems (e.g., disruption of family and community social functioning), exosystems (e.g., intergroup conflict, reduced access to health services), and macrosystems (e.g., social breakdown, forced migration; Noffsinger et al., 2012; Sanson et al., 2019). Risks factors can operate additively, interactively, and cumulatively within and across multiple levels, cascading across development to increase mental-health vulnerability (Masten & Cicchetti, 2010).

**Fig. 2.**
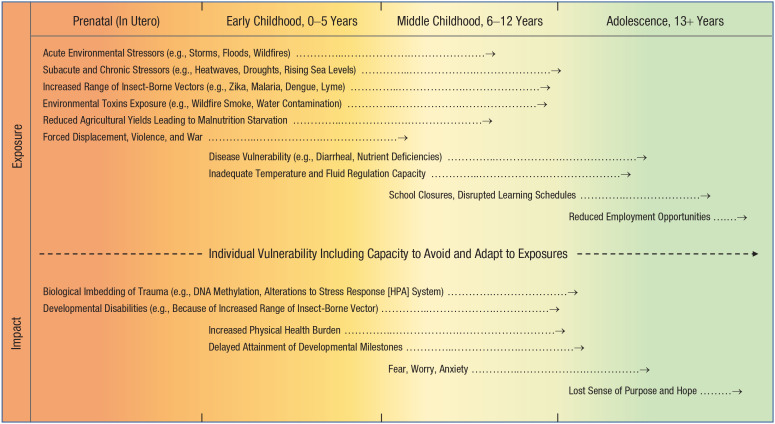
Examples of climate-change-related threats to mental health drawn from the empirical literature. This simplified model shows that climate-change-related exposures can begin before birth (in utero) and have cascading effects across development. Stressors that are severe and protracted and occur early in development typically have larger detrimental effects on development. This is because stressors can operate with additive, interactive, and cumulative effects to increase psychopathology risk both contemporaneously and across time. For climate-change-related risks pertaining to adult mental health, including the dynamic interplay between them, see Berry et al. (2018). For a review of factors linked to healthy child development, see O’Connell et al. (2009). Note that although the Intergovernmental Panel on Climate Change estimates “with high confidence” that there will be a further 1.5 °C increase in average global surface temperatures between 2030 and 2050, predicting future risk embodies inherent uncertainties. Consequently, under different future climate-change scenarios, the importance of specific risks described here may vary, and much will depend on the capacity to mitigate, modify, and adapt to known and emerging risks.

Figure 2 illustrates how climate-change-related exposures (stressors) and their corresponding developmental impacts can lead to increased mental-health vulnerability through multiple pathways from conception onward. Development is divided into four periods: (a) prenatal, (b) early childhood, (c) middle childhood, and (d) adolescence, according to both biological boundaries (e.g., birth, puberty) and socially defined transitions, such as entry to formal education. Some risks may occur within single developmental periods only (e.g., changes to the stress response system due to prenatal stress exposure), whereas others will run across multiple periods, and the effects differ depending on the timing, severity, and chronicity of exposures.

### Prenatal Period

The prenatal period is a life phase of extremely high developmental vulnerability (Olson & Metz, 2020). Climate change will exacerbate acute, subacute, and chronic stressors that harm the developing fetus, illustrated at the top left of Figure 2. Exposures during this period will operate predominantly through biological pathways that alter healthy neurological and physiological growth, whereas psychosocial stressors are mediated by parental and family functioning (microsystem).

Extreme heat exposure is one of the most intuitive and well-documented consequences of global climate change (Fischer et al., 2021). Of the 20 warmest years on record, 19 have occurred since the year 2000 (U.S. National Aeronautics and Space Administration, 2020). Heat waves and hotter average temperatures increase health morbidity (Ebi et al., 2021) and are associated with increased risk of obstetric complications, preterm birth, and stillbirth (Barreca & Schaller, 2020; Bekkar et al., 2020; Poursafa et al., 2015). Obstetric complications are an established risk factor for several major neurodevelopmental and psychiatric disorders and can also affect typical psychological development in the absence of psychiatric diagnosis (Dalman et al., 1999; Glasson et al., 2004). Malaspina et al. (2020) noted that

if prenatal temperature extremes are associated with offspring brain function and behavior and coincide with other prenatal adversities, the future capital of the population could be compromised by more cognitive incapacity and social dysfunction and lower educational attainment, even without psychiatric diagnoses. (p. 778)

Severe weather events are another well-documented pathway through which climate change will affect psychological development (Gamble et al., 2016). They cause widespread damage and destruction that can traumatize pregnant mothers and limit access to nutrition, health care, and physical and psychological safety and subvert development by altering fetal biochemistry and cellular physiology (Charil et al., 2010; Schmitt et al., 2014). Exposure to severe stress during the in utero period has been repeatedly linked with dysregulation of the hypothalamic–pituitary–adrenal (HPA) axis stress response system (Faravelli et al., 2012), whereas exposure to even transient environmental stressors is associated with epigenetic changes, including increased disease vulnerability, that persist throughout life (Aristizabal et al., 2020; Waterland & Michels, 2007). Children exposed to extreme prenatal stress have increased risk for anxiety, mood, behavioral, psychotic, and autism spectrum disorders (Class et al., 2014; Lupien et al., 2009; Markham & Koenig, 2011; Yehuda et al., 2008). Even if exposure to environmental stressors does not result in the onset of a psychiatric disorder, it can lead to delays in attainment of cognitive, behavioral, and socioemotional milestones, which are known to covary with mental-health risk (Evans, 2019; Laplante et al., 2004).

More broadly, slow-moving psychosocial stressors driven by climate change, such as economic downturns, reduced agricultural yields, forced migration, and civil conflict, can have direct effects on maternal stress and, thus, pregnancy outcomes (Olson & Metz, 2020). If persistent, these stressors will continue to undermine the child’s healthy development after the birth, initially mediated by parents’ resources and the quality of parenting they can deliver (Xerxa et al., 2021) and, later, directly through psychosocial stressors within the family (microsystem), educational (mesosystem), and societal (exosystems and macrosystems) environments that the child occupies (R. Williams et al., 2021).

### Early Childhood

Early childhood (0–5 years) is a period of high vulnerability because of physiological immaturity and extremely rapid neuropsychological development. Children form close emotional attachments to caregivers in the first year, followed by rapid language and cognitive development and growing social skills. Malnutrition, dehydration, insect-borne vectors, and air-, water-, and food-borne toxins pose significant risks (Sheffield & Landrigan, 2011). They can directly alter healthy cognitive maturation through biological pathways and through long-term physical health problems that delay the attainment of developmental milestones that interact with psychopathology risk (Firth et al., 2019). Once established, maladaptive developmental trajectories lead to the accumulation of negative life events, such as low academic attainment, unemployment, and welfare reliance (Butler et al., 2014), which enhance psychosocial stress, erode mental-health resilience, and undermine human capital within individuals and across societies (Caspi et al., 1998; Mani et al., 2013).

Indirect (distal) climate-change-related stressors can lead to chronic stress that undermines parents’ resources and resilience and drive poor health behaviors (e.g., poor diet, inadequate physical activity, substance misuse) that directly harm the child and undercut parenting quality; large-scale disruptions can lead to food shortages, child-care closures, and reduced access to health services, further undermining healthy development (Smith & Pollak, 2021). Throughout the early childhood period, children have little control over the psychosocial environment of the family (microsystems) and education contexts (mesosystems), and many threats are mediated by the resourcefulness and responsiveness of parents (Berry et al., 2018).

### Middle Childhood

The middle childhood period (6–12 years) remains a period of high developmental vulnerability. In addition to stressors present during the prenatal or early childhood periods that may be carried over, middle childhood is characterized by new risks arising from growing psychosocial independence, including the formation of social relationships with peers, teachers, and the wider community, which become vulnerable to disruption. Exposure to acute climate-change-related stressors—such as storms, floods, and wildfires—remains a primary pathway to increased psychopathology risk, including attachment problems, disrupted sleep, PTSD, substance use, depression, and anxiety disorders (Clemens et al., 2022; Dean & Stain, 2010; Garcia & Sheehan, 2016; Goldmann & Galea, 2014; Noffsinger et al., 2012; Norris et al., 2002; Simpson et al., 2011). (Studies of childhood PTSD following disasters rarely distinguish between childhood exposure and adolescent exposure.) Among children exposed to disasters, prevalence rates for PTSD, for example, range from 15% to 30% (Alisic et al., 2014); roughly half remain traumatized and symptomatic 18 months later (McDermott et al., 2014). Furthermore, reviews of the disaster literature have found that children experience higher rates of severe mental-health impairments compared with adults (29.6% vs. 18.3%; Norris et al., 2002) and may be more vulnerable than are adults to storm-related PTSD (Stanke et al., 2012).

More broadly, subacute and chronic climate-change-related stressors—such as heat stress, droughts, reduced agricultural yields, and economic downturns—may have flow-on effects that disrupt educational attainment, leisure activities, and social-support networks (Carnie et al., 2011). Once again, these disruptions can delay attainment of developmental milestones, interfere with the healthy transition to adolescence, and increase mental-health vulnerability (Akresh, 2016; Garcia & Sheehan, 2016). During the middle-childhood period, fear of accidents and catastrophes is common and developmentally typical, and worry about climate change and its anticipated effects may increase.

### Adolescence

Adolescence is characterized by major physiological, neurocognitive, and hormonal changes, and the appearance of new psychiatric disorders peaks at this time (Solmi et al., 2022). These changes are accompanied by increasing psychosocial independence, including the formation of a more stable personal identity, peer groups, leisure activities, and increasing self-reliance. Adverse impacts arising from exposure to severe weather events remain high, particularly when disruptions affect social-support networks (Hinton et al., 2019). Heat waves and higher ambient temperatures undermine sleep quality (Minor et al., 2020), learning (Park et al., 2021), cognitive-test performance (Park, 2022), and high school graduation rates (Graff Zivin et al., 2017). These outcomes can impede the timely attainment of educational milestones and curtail future potential for full social and economic participation in society. Adolescents show growing awareness of abstract values, including justice, responsibility, and human rights, and increasing interest in civic and economic participation (exosystems and macrosystems) and, even more than children, are likely to understand the causes and consequences of climate change (Box 2).

Data drawn from international samples show that children and adolescents are increasingly aware of and worried about climate change (Lee et al., 2020). This was starkly highlighted in the 2018 school strikes initiated by Swedish school student Greta Thunberg that took place in at least 123 countries (Marris, 2019). Worry about climate change can engender feelings of despair and helplessness that aggravate symptoms for existing disorders and lower the threshold for the onset of new ones, particularly among children living in rural and remote locations (Carnie et al., 2011). Although this idea has been mooted in relation to mental health of adults (Clayton, 2020; Cunsolo & Ellis, 2018) and children (Majeed & Lee, 2017), empirical support is limited, and it remains fertile ground for future work.

In summary, changing weather- and climate-related events are driving new threats to human health. These stressors, particularly when severe, protracted, and occurring early in development, have cascading effects, dynamically spreading risks across biological and psychosocial systems to undermine healthy psychological development. Developmental psychopathology provides an integrative and interdisciplinary framework for conceptualizing these disparate risks and guiding research across academic disciplines so that they can be monitored and mitigated. Below we describe some conceptual and measurement challenges, including how emerging threats can be tracked and measured.

## Conceptual and Measurement Challenges

Studies of climate-change-related exposures and mental-health outcomes of children remain scarce, especially over the long term (Magalhaes et al., 2020; Berrang-Ford et al., 2021; Helldén et al., 2021). There is a need for well-controlled studies that describe the pathways and quantify the effects of acute (e.g., floods, wildfires), subacute (e.g., heat waves), and chronic stressors (e.g., droughts, food shortages) on psychological development and mental-health vulnerability. The mechanisms underlying these effects require further study as the basis for intervention planning and evidence-based policy development—for example, whether the association between heat exposure and psychological distress is explained by reduced sleep quality (Obradovich & Migliorini, 2018). Moderating (protective; Mah et al., 2020) and promotive factors (Zimmerman et al., 2013) and the mechanisms underlying resilient functioning (Cicchetti, 2010) should also be evaluated to inform adaptation and prevention planning.

Although human and animal studies have repeatedly linked early adverse life events to negative developmental outcomes, the mechanisms underlying these effects remain poorly understood and are thought to be cumulative, nonspecific, and unlinked to particular categories of psychopathology (Woodard & Pollak, 2020). Attention should therefore be paid to questions of intensity, severity, chronicity, and developmental timing of climate-change-related stressors (Fernandez et al., 2020). For instance, recent work suggests that adverse experiences in adolescence can have larger negative effects on future human-capital formation (including mental health) than equivalent experiences that occur in earlier childhood periods (Andersen, 2021). This challenges the “first 1,000 days” narrative of a unique period of developmental sensitivity and underscores the need to examine other potentially sensitive periods (Huei-Jong et al., 2021).

When possible, studies should rely on objective measures of exposure, such as meteorological records and standardized measures of psychopathology and psychological well-being obtained from administrative data (e.g., medical records) to facilitate comparisons across studies and populations. Although the limitations of using self-report data have been extensively debated, these data are not necessarily “second best” (Idler & Benyamini, 1997), particularly in cases in which measures have demonstrably strong psychometric properties and individuals’ perceptions and opinions are germane, as is the case with subjective well-being (Lucas, 2018). The interplay between subjective and so-called objective measures should be considered in measurement planning (Chang et al., 2020). For instance, children’s subjective perceptions of traumatic life events can be more predictive of adult life psychopathology than “objective” measures (Danese & Widom, 2020). Males and females differ in their vulnerability to neuro-psychiatric disorders and to noncommunicable disease risk, and the gendered element of climate-change risk should be considered in research and response planning (Nelson et al., 2002).

Despite mounting public discourse about the psychological effects of climate change, few empirical studies have addressed the question of how children and adolescents are responding to climate change as a stressor, the feelings elicited by it, the effects of worry on their psychological well-being, and what they are doing to cope (Burke et al., 2018). A handful of studies have examined children’s and adolescents’ psychological adaptation and coping styles among Swedish school students (Ojala, 2012; Ojala & Bengtsson, 2019), but more work is needed to examine effects across developmentally sensitive periods and more diverse populations and contexts.

Overall, there is very little empirical research on climate change, child development, and mental health and, thus, much work to be done (Berrang-Ford et al., 2021). This requires practical solutions within a robust and inclusive propositional framework. Consequently, we advocate taking a developmental perspective situated within a broader systems-thinking approach because it allows complexity, subtlety, and accounting for the long and interactive causal chains that characterize the development and outcomes of psychological health and well-being (Berry et al., 2018). From there, we aim to leverage the best available measurement opportunities, among which, birth-cohort studies and administrative data appear particularly promising because they can yield the quantity and, particularly, the quality of data needed. Furthermore, birth cohorts can be immediately leveraged by including climate-change-relevant measures right now so that their richness can be contemporaneously used and, over time, fully exploited (Box 3).

**Box 3**

**Leveraging Birth-Cohort Studies**

Cohort studies, and particularly birth-cohort studies, are well suited to studying the effects of complex short- and long-term exposures arising from climate change. Their strength lies in several key features, including sample size, sampling strategy, exposure measurement, repeated assessment, and phenotypic richness. This makes it possible to test key questions about the onset and course of psychopathology that cannot easily be evaluated using other research designs (Kordas & Park, 2015). In the context of climate change, birth-cohort studies offer several key advantages:

1. Long-term tracking: Assessment can begin before birth and span the most important life phases for psychosocial development while tracking climate-change-related threats and exposures across time. Studies may be extended well into adulthood, providing the opportunity to gauge the long-term effects of childhood exposures on development and psychopathology.
2. Confounding control: The prospective design, large sample size, measurement richness (e.g., biological, psychological, social), and robust control for background factors (e.g., socioeconomic status) that covary with mental-health risk allow stronger causal inferences to be made (VanderWeele, 2021). This makes them valuable for policy development.
3. Explanatory pathways: The prospective repeated measures design allows competing and complementary processes to be tested, including mediating and moderating mechanisms, which can aid the development of prevention and intervention programs. Potential benefits arising from climate change, such as increased social cohesion in response to mitigation efforts, can also be examined.
4. Flexible design: With measurement infrastructure already in place, climate-change-relevant items can be incorporated into existing birth-cohort studies without having to wait years for new data to be collected. Birth-cohort studies can be readily leveraged, for instance, through the inclusion of randomized trials, brief cross-sectional analyses (e.g., children’s psychological responses to climate change), “natural experiments” following exposure to environmental stressors, and genetic data (Pingault et al., 2018).
5. Data linkages: Birth cohorts can be linked with administrative data such as medical, educational, criminal justice, census, taxation, and meteorological records (e.g., heat, humidity, precipitation, storm severity, and air quality) and thus enhance their quality and reach (e.g., because of low attrition rates, population-wide coverage, and high reliability).
6. Investment in the future: Birth-cohort studies reflect present-day values and priorities but also the opportunity for investment in the health and well-being of future generations. Introducing relevant climate-change-related metal health measures into birth-cohort studies will encourage conceptual and methodological development and bring attention—including research funding—to this neglected area (Green et al., 2017).

From a measurement point of view, direct threats are more tangible and visible than indirect threats, which makes them easier to evaluate and mitigate and, thus, to gain support from government bodies, funding agencies, health care services, and the public. Although we acknowledge the limitations of overemphasizing proximal threats (Berry et al., 2018; McMichael, 1999), we must start where we feasibly can and suggest an *initial* focus on objectively measured direct (proximal) climate-change-related exposures in conjunction with subjective assessments of psychological responses to traumas (see Table S1 in the Supplemental Material available online). A more inclusive list of research priorities is given in Box 4.

**Box 4**

**Priority Research Areas**

- Review and synthesize literature on the effects of climate-change-related events on healthy psychological development from the prenatal period to adulthood (and, eventually, old age)
- Conduct rigorous interdisciplinary research on the effect of acute (e.g., wildfires, floods), subacute (e.g., heatwaves), and chronic (e.g., drought, food shortages) stressors on the cognitive, social, and emotional development of children across the world
- Examine how the intensity, severity, chronicity, and developmental timing of exposures influence the healthy psychological development of children (including additive, interactive and cumulative effects)
- Clarify the mechanisms through which climate-change-related stressors increase mental-health vulnerability (e.g., disrupted sleep, lost education)
- Conduct cross-cultural studies on how children are adapting psychologically to climate change (e.g., fear, anxiety) and what they are doing to cope
- Evaluate how resilience can be promoted and vulnerability reduced at the individual and, especially, the group levels so that children are better equipped to live with climate change
- Identify existing and new research designs, analytic approaches, and data sources that can help address these questions in the immediate and long term

## Participation and Prevention

Climate change is now a pressing child-rights challenge (UNICEF, 2021). As a major stakeholder group—the world’s future adults, no less—children have a right to participate in climate-change discourse and decision-making at the local and global levels (Vaghri, 2018). Children can play a key role in adaptation and disaster risk reduction and should be empowered to do so (Seddighi et al., 2020) by harnessing their energy, optimism, and enthusiasm to work for a greener future of their own design (Berry, 2021). Effective climate-change education is key to empowerment. A recent review called for “the development of new forms of climate change education that directly involve young people in responding to the scientific, social, ethical, and political complexities of climate change” (Rousell & Cutter-Mackenzie-Knowles, 2020, p. 191), and further work to establish age-appropriate “best practice” for improving young people’s engagement with climate change has been called for (Mah et al., 2020).

Children from low- and middle-income countries represent some 85% of the world’s children. They are at once disproportionately affected by climate change and least empowered to participate in its solutions and, thus, must be included in research and adaptation planning. Currently, most of the knowledge about the mental-health impacts of climate change has been generated in the Global North using data drawn from samples in Western, educated, industrialized, rich, and democratic societies, especially North America (Berrang-Ford et al. 2021), and is therefore not representative of the global majority (Henrich et al., 2010). These inequalities must be acknowledged by stakeholder groups (e.g., governments, health agencies, researchers) so that locally adapted, culturally informed measurement and mitigation efforts can be implemented (Zhang et al., 2021).

Climate change will be a key driver of existing mental-health inequalities both within and between nations, and identifying vulnerable populations should be a priority. This includes children with existing mental-health difficulties, those living in rural and socially isolated communities with high social and health inequalities, those who depend on the land directly for survival (e.g., farming communities, indigenous populations) and who live in vulnerable regions (e.g., flood, drought, and fire prone) and, especially, those living in low- and middle-income countries that lack the resources to effectively respond to climate change threats and impacts. Interventions for specific psychiatric disorders arising in the context of climate change-related stressors should follow best-practice clinical guidelines. The psychological distress associated with the existential threat of climate change—such as worry, anxiety, and depression—may also need to be addressed (Taylor, 2020). Unfortunately, several recent meta-analyses have shown that prevention programs for anxiety and mood disorders in children and youth yield small or weak effects (Caldwell et al., 2019; Moreno-Peral et al., 2019) and further development and refinement of current approaches is necessary. At the population level, it may be possible to improve mental-health resilience by harnessing family and community support (“social prescribing”), improving climate-change awareness and support capacity of primary health services, and reducing social and health inequalities (Cunsolo et al. 2020; Fusar-Poli et al., 2021). School-based psychoeducation programs that teach children about climate change and mental-health risks alongside solution-focused coping strategies may be effective but have yet to be evaluated. These programs should be culturally sensitive, regionally specific, and rigorously tested before roll-out.

More broadly, we strongly favor community-based actions because they shift the onus from primarily the individual to primarily the group. This is the direction that the field needs to go, from both a mental-health promotion and prevention and a treatment and rehabilitation perspective. We suggest a model of “preventive psychiatry” that prioritizes investment in universal public-health approaches targeting the social determinants of mental disorders (Fusar-Poli et al., 2021). This requires increased investment in education, employment, social support, housing, criminal justice, poverty alleviation, community development, greenspace, environmental protection, and immigration reform. The approach is compatible with the global mental-health and sustainable development goals (Patel et al., 2018) that seek to improve global mental health while simultaneously tackling climate change and protecting the environment.

## Summary and Conclusions

Healthy psychological development underpins the future capital of society and, thus, its capacity to evolve quickly and intelligently. Climate change is undermining this capital. Risks begin before birth and cascade across development; they are already being observed and are projected to increase in coming decades. Most countries are inadequately prepared and underresourced to respond to these challenges. Young people today will bear the long-term burden of living with and “solving” climate change despite being least responsible for causing it, and swift and effective action to minimize this burden is therefore a matter of international and intergenerational justice as well as a substantive practical imperative. This includes doing much more to understand—theoretically and empirically—how climate change influences psychological health and well-being from conception to adulthood and the mechanisms through which effects emerge, interact, and proliferate. It is essential for effective adaptive and mitigation planning and a vital investment in the mental health and well-being of children, society, and future generations.

## Supplemental Material

sj-pdf-1-cpx-10.1177_21677026211040787 – Supplemental material for Climate Change and Children’s Mental Health: A Developmental Perspective Click here for additional data file. Supplemental material, sj-pdf-1-cpx-10.1177_21677026211040787 for Climate Change and Children’s Mental Health: A Developmental Perspective by Francis Vergunst and Helen L. Berry in Clinical Psychological Science

## Transparency

*Action Editor:* Steve S. Lee

*Editor:* Jennifer L. Tackett

*Author Contributions*

F. Vergunst and H. L. Berry conceptualized the study. F. Vergunst drafted the manuscript. Both authors contributed to revision and approved the final manuscript for submission.
