# Supplementary material for: Climate Change and Children’s Mental Health: A Developmental Perspective
Source: Clin Psychol Sci. 2021 Sep 14;10(4):767–85. doi: 10.1177/21677026211040787 (PMC9280699; doi:10.1177/21677026211040787)
Supplement: sj-pdf-1-cpx-10.1177_21677026211040787 – Supplemental material for Climate Change and Children’s Mental Health: A Developmental Perspective [file sj-pdf-1-cpx-10.1177_21677026211040787.pdf]

Climate change and children's mental health – A developmental perspective

Francis Vergunst, DPhil and Helen L Berry, PhD

University of Montreal, Montreal, Quebec, Canada (Vergunst)
Australian Institute of Health Innovation, Macquarie University, Sydney, Australia (Berry)

Correspondence to Francis Vergunst (francis.vergunst@umontreal.ca) Ste-Justine Hospital Research Center, 3175, Côte Sainte-Catherine, Étage A, Local A-568, Montréal (Québec) Canada, H3T 1C5. Tel: 1-514 345-2182, Fax: 514 345-2176

TABLE S1: Exposure to severe weather events and its impact on psychological health and wellbeing

**Item**	**Scoring**	**Derivation**
"Were you affected in any way by a weather-related disaster (e.g., flood, wildfire, storm, cyclone) in the past 12 months?"	Response on a scale of 1 to 10, where 1 is not at all and 10 is very severely	Berry, Adapted from the HILDA survey and O'Donnell (2008)
"In the last 12 months, did a weather-related disaster (e.g., flood, wildfire, storm, cyclone) severely damage or destroy:<br>(a) your home?<br>(b) your suburb or local area?<br>(c) the home of a family member or friend?	For each item endorsed, ask:<br>• On a scale of 1 to 10, where 1 is not at all and 10 is very severely, how badly were you affected by [your suburb etc] being flooded?	Berry, Adapted from O'Donnell (2008)
"Did any of the following happen as a result of this weather-related disaster?" YES NO<br>(a) You are worried about how you will manage<br>(b) You thought you might be badly injured or even die<br>(c) You personally knew people who were killed or badly injured<br>(d) You felt terrified, helpless or hopeless<br>(e) You had to move out of your home (even briefly)<br>(f) You are still <u>currently</u> distressed about it<br>(g) You received professional help or support for your distress	If item (a) endorsed, ask:<br>• How often are you worried about how you will manage?<br>5-point scale ranging from 1= 'none of the time' to 5= 'all of the time'<br><br>If item (f) endorsed, ask:<br>• How often do you currently have distressing thoughts about the event?<br>5-point scale ranging from 1= 'none of the time' to 5= 'all of the time'	Berry, Adapted from O'Donnell (2008)<br>"You felt terrified, helpless or hopeless" (Consistent with diagnostic criteria (A2) for PTSD (DSMIV) and ICD entry criteria).<br>"You are still currently distressed about it" (Allows calculation of point prevalence of post-disaster distress and differentiation from other possible causes of anxiety; can be validated against related constructs measured in the cohort. This item provides insight into whether the validated anxiety item is directly related to the traumatic event).
"Were you or a close family member admitted to hospital due to injuries caused by the severe weather event?"	Yes – you=2, family=1, no=0	Berry, Adapted from O'Donnell (2008)
